# Both common variations and rare non-synonymous substitutions and small insertion/deletions in *CLU *are associated with increased Alzheimer risk

**DOI:** 10.1186/1750-1326-7-3

**Published:** 2012-01-16

**Authors:** Karolien Bettens, Nathalie Brouwers, Sebastiaan Engelborghs, Jean-Charles Lambert, Ekaterina Rogaeva, Rik Vandenberghe, Nathalie Le Bastard, Florence Pasquier, Steven Vermeulen, Jasper Van Dongen, Maria Mattheijssens, Karin Peeters, Richard Mayeux, Peter St George-Hyslop, Philippe Amouyel, Peter P De Deyn, Kristel Sleegers, Christine Van Broeckhoven

**Affiliations:** 1 Neurodegenerative Brain Diseases Group, Department of Molecular Genetics, VIB, 2610 Antwerpen, Belgium; 2 Institute Born-Bunge, University of Antwerp, 2610 Antwerpen, Belgium; 3 Department of Neurology and Memory Clinic, Hospital Network Antwerp Middelheim and Hoge Beuken, 2610 Antwerpen, Belgium; 4 INSERM U744, Institut Pasteur de Lille, Université Lille Nord de France, 59019 Lille, France; 5 CHR&U de Lille, Université Lille Nord de France, 59019 Lille, France; 6 Centre for Research in Neurodegenerative Diseases, Department of Medicine (Neurology), University of Toronto, Toronto, Ontario M5S 1A8, Canada; 7 Department of Neurology, University Hospitals Leuven and University of Leuven, 3000 Leuven, Belgium; 8 Gertrude H. Sergievsky Center, Columbia University, New York NY 10032, USA; 9 Cambridge Institute for Medical Research, and the Department of Clinical Neurosciences, University of Cambridge, Cambridge, UK CB2 0XY

**Keywords:** Alzheimer disease, clusterin gene (*CLU*), genomic resequencing, non-synonymous substitutions, insertions/deletions, β-chain domain, meta-analysis

## Abstract

**Background:**

We have followed-up on the recent genome-wide association (GWA) of the clusterin gene (*CLU) *with increased risk for Alzheimer disease (AD), by performing an unbiased resequencing of all *CLU *coding exons and regulatory regions in an extended Flanders-Belgian cohort of Caucasian AD patients and control individuals (*n *= 1930). Moreover, we have replicated genetic findings by targeted resequencing in independent Caucasian cohorts of French (*n *= 2182) and Canadian (*n *= 573) origin and by performing meta-analysis combining our data with previous genetic *CLU *screenings.

**Results:**

In the Flanders-Belgian cohort, we identified significant clustering in exons 5-8 of rare genetic variations leading to non-synonymous substitutions and a 9-bp insertion/deletion affecting the CLU β-chain (*p *= 0.02). Replicating this observation by targeted resequencing of *CLU *exons 5-8 in 2 independent Caucasian cohorts of French and Canadian origin identified identical as well as novel non-synonymous substitutions and small insertion/deletions. A meta-analysis, combining the datasets of the 3 cohorts with published *CLU *sequencing data, confirmed that rare coding variations in the CLU β-chain were significantly enriched in AD patients (OR_MH _= 1.96 [95% CI = 1.18-3.25]; *p *= 0.009). Single nucleotide polymorphisms (SNPs) association analysis indicated the common AD risk association (GWA SNP rs11136000, *p *= 0.013) in the 3 combined datasets could not be explained by the presence of the rare coding variations we identified. Further, high-density SNP mapping in the *CLU *locus mapped the common association signal to a more 5' *CLU *region.

**Conclusions:**

We identified a new genetic risk association of AD with rare coding *CLU *variations that is independent of the 5' common association signal identified in the GWA studies. At this stage the role of these coding variations and their likely effect on the β-chain domain and CLU protein functioning remains unclear and requires further studies.

## Background

Genome-wide association (GWA) studies lead to long-awaited breakthroughs in the genetics of late-onset Alzheimer disease (AD) [MIM 104300] [1] by providing conclusive genetic association evidence for novel AD risk genes [2-5]. Notably, GWA significance with similar effect sizes was reached for the top single nucleotide polymorphism (SNP) rs11136000 in the clusterin gene (*CLU*) [MIM 185430] [1]. The CLU protein (also known as apolipoprotein J) is a multifunctional protein showing functional similarities with the major apolipoprotein of the brain, apolipoprotein E (APOE) [6]. In relation to AD, CLU expression is increased in pyramidal neurons and astrocytes of the hippocampus and entorhinal cortex, the most severely affected brain regions in AD [7]. CLU is present in senile plaques [8], binds to Aβ and is involved in Aβ_42 _clearance across the blood brain barrier [9]. Moreover, CLU enhances endocytosis of Aβ aggregates to brain phagocytes [10]. Taking its variety of physiological functions, CLU could be a guardian or enemy in AD [11].

**Figure 1:**
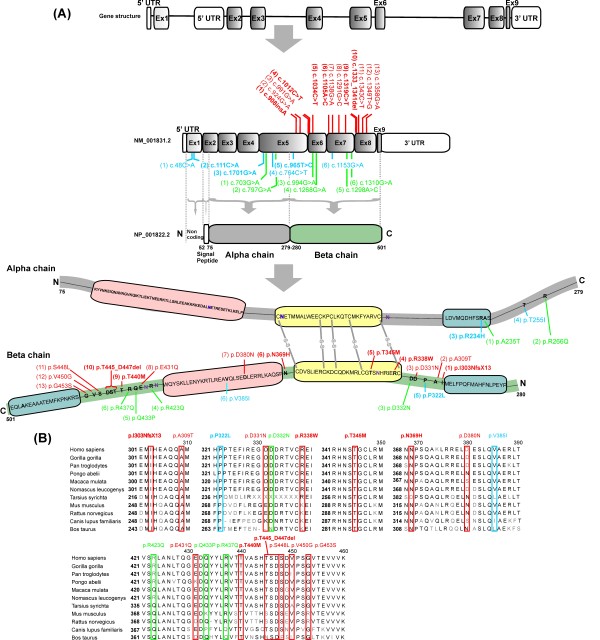
**Schematic location of rare *CLU *coding variants identified in stage I, II and III resequencing**. **(A) **Schematic presentation of *CLU *gene structure, CLU transcript 1 [NM_001831.2] and CLU protein [NP_001822.2]. Coding variants observed in AD patients only are indicated in red, variants observed in patients and controls in blue, variants detected in control individuals only in green. All predicted pathogenic variants are indicated in bold. After cleavage of the signal peptide, the secreted CLU form (449 AA) contains two coiled-coiled domains (pink), three amphipathic domains (blue) and a cysteine rich region (yellow) with 5 disulfide bridges (grey). Six N-glycosylation sites are marked in purple. For ease of interpretation, amino acids are given for specific CLU domains and for detected protein variants only. **(B) **Conservation alignment of amino acids of CLU beta-chain variants is shown in different species; Homo sapiens (ENSP00000315130), Gorilla gorilla (ENSGGOP00000016521), Pan troglodytes (ENSPTRP00000034423), Pongo abelii (ENSPPYP00000020696), Macaca mulata (ENSMMUP0000003216), Nomascus leucogenys (ENSNLEP00000020015), Tarsius syrichta (ENSTSYP00000001230), Mus musculus (ENSMUSP00000022616), Rattus norvegicus (ENSRNOP00000022095), Canis lupus familiaris (ENSCAFP00000012350) and Bos taurus (ENSBTAP00000007324). Similar to panel A, patient specific variants are marked in red, variants observed in patients and controls in blue and variants in control individuals in green. All predicted pathogenic variants are marked in bold.

The *CLU *transcriptional unit is located in the chromosomal region 8p21-p12 and comprises 9 exons in the longest transcript that translates in the main CLU protein isoform of a 449 amino-acid residues. The CLU precursor peptide is internally cleaved to produce an α- and β-subunit, held together by disulphide bridges and is subsequently secreted from the cell (Figure 1)[12].

In this follow-up study aiming at identifying the genetic variant underlying the *CLU *association with increased AD risk, we examined the *CLU *genetic variability using a resequencing approach including all coding exons and regulatory regions in a well-documented Flanders-Belgian patient/control cohort (*n *= 1930 subjects) [2,3]. Significant genetic findings were verified by targeted resequencing in two independently ascertained French [3] and Canadian [13] AD cohorts and by meta-analyses of the genetic data sets generated in this study with published data sets obtained of previous genetic screenings of *CLU *in AD cohorts [14,15].

## Results

### *CLU *resequencing

The stage I resequencing of the coding exons and the regulatory regions of *CLU *in patients and control individuals of the Flanders-Belgian AD cohort (*n *= 1930) (Table 1), identified in total 19 rare to intermediate rare non-synonymous single nucleotide variations predicting an amino acid substitution in the CLU protein of which only 5 had been reported earlier [14,15] (Table 2). Further, we detected an in-frame 9-bp deletion predicting a 3 amino acid deletion p.T445_D447del. Fourteen of the 19 non-synonymous substitutions occurred in 31 AD patients (*n *= 849, 3.6%), of which 8 appeared only in patients (*n *= 11), and 6 in patients (*n *= 20) and control individuals (*n *= 20). One AD patient carried 2 non-synonymous substitutions p.R338W and p.T345M, and one healthy individual carried both p.S16R and p.R234H. All 3 AD patients with p.T445_D447del carried also p.A309T. The remaining 5 non-synonymous substitutions occurred only in control individuals (*n *= 5), adding up to 25 control carriers (*n *= 659, 3.8%) (Table 2 Figure 1).

**Table 1:** Description of cohorts.

	Stage I: Flanders-Belgian AD cohort	Stage II: Lille AD cohort	Stage II: Toronto AD cohort	Stage III: Caribbean Hispanic AD cohort
Total number of subjects	1930	2182	573	1045

AD patients	1057	1465	323	520
Mean AAO (years) (SD)	74.9 ± 8.9	69.5 ± 8.2	75.3 ± 9.7	79.7 ± 8.6
Women (%)	692 (65.5)	969 (66.1)	178 (55.1)	369 (71.0)
At least one *APOE *ε4 allele (%)	565 (53.5)	852 (58.2)	182 (56.3)	207 (39.9)

Control individuals	873	717	250	525
Mean AAI (years) (SD)	65.1 ± 14.9	74.0 ± 8.0	73.0 ± 10.2	78.9 ± 6.5
Women (%)	501 (57.4)	447 (62.3)	149 (60.0)	358 (68.2)
At least one *APOE *ε4 allele (%)	235 (26.9)	145 (20.2)	60 (24.0)	126 (24.0)

AAO = age at onset; SD = standard deviation; AAI = age at inclusion.

**Table 2:** Rare non-synonymous *CLU *variants identified in Stage I Flanders-Belgium AD cohort.

Stage I: Flanders-Belgium AD cohort									
Gene location^a^	DNA^b^	Protein^c^	dbSNP	Total number	MAF AD	MAF C	Protein location	PolyPhen (PSIC)	SIFT
Exon 5	c.924G > A	p.A309T		3	0.0018 ^(1)^		β-chain	benign (1.07)	tolerated (0.65)
Exon 6	c.1012C > T	p.R338W		2	0.0012 ^(2)^		β-chain	probable (2.37)	not tolerated (0.00)
Exon 6	c.1034C > T	p.T345M		1	0.0006 ^(2)^		β-chain	possible (1.56)	tolerated (0.09)
Exon 7	c.1105A > C	p.N369H	rs9331936	2	0.0012		β-chain	possible (1.56)	tolerated (0.15)
Exon 7	c.1138G > A	p.D380N	rs9331938	1	0.0006		β-chain	benign (0.32)	tolerated (0.36)
Exon 7	c.1319C > T	p.T440M		1	0.0006		β-chain	possible (1.75)	not tolerated (0.01)
Exon 8	c.1333_1341del	p.T445_D447 del		3	0.0018 ^(1)^		β-chain		
Exon 8	c.1343C > T	p.S448L	rs13494	1	0.0006		β-chain	benign (0.73)	tolerated (0.23)
Exon 8	c.1349T > G	p.V450G		1	0.0006		β-chain	benign (1.42)	tolerated (0.15)

Exon 1	c.48C > A	p.S16R		11	0.0029 (5 AD)	0.0046 (6 C) ^(3)^	extra AA isoform 2	benign (1.13)	not tolerated (0.00)
Exon 1	c.111C > A	p.H37Q		4	0.0012 (2 AD)	0.0015 (2 C)	extra AA isoform 2	possible (1.80)	not tolerated (0.00)
Exon 5	c.701G > A	p.R234H		3	0.0012 (2 AD)	0.0008 (1 C) ^(3)^	α-chain	possible (1.64)	tolerated (0.15)
Exon 5	c.764C > T	p.T255I	rs4127629	11	0.0029 (5 AD)	0.0046 (6 C)	α-chain	benign (0.31)	tolerated (0.29)
Exon 5	c.965T > C	p.P322L		9	0.0029 (5 AD)	0.0030 (4 C)	β-chain	possible (1.96)	tolerated (0.25)
Exon 7	c.1153G > A	p.V385I		3	0.0006 (1 AD)	0.0015 (2 C)	β-chain	benign (0.15)	tolerated (0.40)

Exon 5	c.703G > A	p.A235T		1		0.0008	α-chain	benign (1.18)	tolerated (0.16)
Exon 5	c.797G > A	p.R266Q		1		0.0008	α-chain	benign (0.27)	tolerated (0.67)
Exon 6	c.994G > A	p.D332N		1		0.0008	β-chain	benign (0.04)	tolerated (0.1)
Exon 7	c.1268G > A	p.R423Q		1		0.0008	β-chain	benign (0.33)	tolerated (0.54)
Exon 7	c.1298A > C	p.Q433P		1		0.0008	β-chain	benign (0.41)	tolerated (0.13)

^a^Gene location position according to the longest *CLU *transcript with 9 coding exons [NM_001831.2], ^b^Numbering according to CLU mRNA sequence starting at the A of the ATG translation initation codon in the reference sequence [GenBank accesion number NM_001831.2], ^c^Numbering according to CLU protein sequence [GenPept accession number NP_001822.2]; MAF = Minor Allele Frequency, calculated upon the minimum number of successful sequences (1698 patient and 1318 control alleles); ^(1) ^3 AD patients carry 2 different *CLU *variants ^(2) ^1 AD patient with 2 *CLU *variants ^(3) ^1 control individual with 2 *CLU *variants. Prediction of pathogenicity of missense mutations was performed using *in silico *programs PolyPhen (benign/possibly damaging/probably damaging) and SIFT (tolerated/not tolerated).

Seven of the 14 non-synonymous substitutions observed in patients were predicted to have a possible or probable harmful effect on CLU protein structure and function using physical and comparative considerations embedded in the PolyPhen software [16](Table 2). SIFT software [17,18] assessing whether the amino acid substitutions had an effect on CLU protein function based on evolutionary conservation identified 3 non-tolerated substitutions (p.R338W, p.S16R and p.H37Q) (Table 2) (Figure 1). For 4 patients carrying a rare non-synonymous *CLU *variant, we obtained positive disease history data. In their pedigree, we identified only one other affected first degree relative which suggested that these rare variants have an intermediate disease penetrance.

In contrast, all 5 non-synonymous substitutions detected only in control individuals were labeled by PolyPhen software as benign and were tolerated according to SIFT software (Table 2 Figure 1).

Genetic variations in regulatory elements were also detected in AD patients but none of these genetic variants were located within conserved transcription factor binding sites (See table part D in Additional file 1). In addition, no *CLU *variants were found affecting splicing using FSPLICE, Netgene2 and SPL prediction programs (See table part E in Additional file 1).

### Association analyses of rare *CLU *variants

Though overall the percentages of *CLU *rare variant carriers among AD patients and control individuals was similar, visible inspection of the distribution of the non-synonymous substitutions in AD patients suggested clustering in exons 5-8 which for the most part are coding for the CLU β-chain domain (Table 2) (Figure 1). Statistical analysis confirmed significant clustering in exons 5-8 with 3.8 times more carriers of non-synonymous substitutions or a deletion mutation among patients than control individuals (*p *= 0.02). To confirm this observation, we used targeted resequencing of exons 5-8 in two independent replication AD cohorts of French and Canadian origin (*n *= 2755). Again we identified non-synonymous *CLU *substitutions of which p.R338W, p.N369H and insertion/deletion p.T445_D447del that were already observed in Flanders-Belgian patients. Also, in the Lille AD cohort one 1 bp-insertion predicted a frameshift and premature stop codon - p.I303NfsX13 was found in one AD patient (Table 3) (Figure 1).

**Table 3:** Rare non-synonymous *CLU *variants identified in Stage II Lille and Toronto AD cohorts.

Stage II: Lille AD cohort									
Gene location^a^	DNA^b^	Protein^c^	dbSNP	Total number	MAF AD	MAF C	Protein location	PolyPhen (PSIC)	SIFT
Exon 5	c.908insA	p.I303NfsX13		1	0.0004		β-chain		
Exon 6	c.1012C > T	p.R338W		1	0.0004		β-chain	probable (2.37)	not tolerated (0.00)
Exon 7	c.1105A > C	p.N369H	rs9331936	6	0.0023		β-chain	possible (1.56)	tolerated (0.15)
Exon 8	c.1333_1341del	p.T445_D447del		3	0.0011		β-chain		
Exon 8	c.1343C > T	p.S448L	rs13494	1	0.0004		β-chain	benign (0.73)	tolerated (0.23)
Exon 8	c.1358G > A	p.G453S	rs34627536	1	0.0004		β-chain	benign (0.39)	

Exon 5	c.965T > C	p.P322L		5	0.0015 (4 AD)	0.0008 (1 C)	β-chain	possible (1.96)	tolerated (0.25)
Exon 7	c.1138G > A	p.D380N	rs9331938	5	0.0015 (4 AD)	0.0008 (1 C)	β-chain	benign (0.32)	tolerated (0.36)

Exon 7	c.1310G > A	p.R437Q		1		0.0008	β-chain	benign (0.700)	

**Stage II: Toronto AD cohort**									
**Gene location^a^**	**DNA^b^**	**Protein^c^**	**dbSNP**	**Total Number**	**MAF AD**	**MAF C**	**Protein location**	**PolyPhen (PSIC)**	**SIFT**

Exon 7	c.1138G > A	p.D380N	rs9331938	1	0.0016		β-chain	benign (0.322)	tolerated (0.36)
Exon 8	c.1333_1341del	p.T445_D447del		1	0.0016		β-chain		

Exon 7	c.1105A > C	p.N369H	rs9331936	3	0.0033 (2 AD)	0.0022 (1 C)	β-chain	possibly (1.557)	tolerated (0.15)
Exon 8	c.1343C > T	p.S448L	rs13494	3	0.0033 (2 AD)	0.0022 (1 C)	β-chain	benign (0.73)	tolerated (0.23)

^a^Gene location position according to the longest *CLU *transcript with 9 coding exons [NM_001831.2], ^b^Numbering according to CLU mRNA sequence starting at the A of the ATG translation initation codon in the reference sequence [GenBank accesion number NM_001831.2], ^c^Numbering according to CLU protein sequence [GenPept accession number NP_001822.2]; MAF = Minor Allele Frequency, calculated upon the minimum number of successful sequences (Lille cohort: 2650 patient and 1214 control alleles; Toronto cohort: 608 patient and 462 control alleles). Prediction of pathogenicity of missense mutations was performed using *in silico *programs PolyPhen (benign/possibly damaging/probably damaging) and SIFT (tolerated/not tolerated).

Allele sharing among p.T445_D447del carriers i.e. 3 Flanders-Belgian, 3 French, 1 Canadian patients and offspring of two Belgian patients was examined by genotyping short tandem repeat markers located in a 1.3 Mb region of the *CLU *locus (See table in Additional file 2). DNA available of two children (non-affected, inclusion ages < 50 years) allowed to reconstruct phased haplotypes. No other family members were available. The 3 Flanders-Belgian patients shared alleles at 10 neighboring markers constituting a haplotype of 1.3 Mb around *CLU*. Seven of these consecutive markers were shared by 2 French patients. A third French patient and a Canadian patient carrier shared 3 markers of this haplotype near *CLU*. Of note, only Flanders-Belgian patients also carried non-synonymous variant p.A309T.

All non-synonymous substitutions and the deletion/insertion mutations observed in the 3 AD cohorts affecting the CLU β-chain domain were carried forward in a meta-analysis (Table 4). Similarly, we included in the meta-analysis previously reported non-synonymous substitutions that were observed in Portuguese, UK and US Caucasian AD cohorts [14,15]. Combining the information of in total 6724 patient and 4820 control chromosomes, the meta-analysis confirmed that non-synonymous substitutions and insertion/deletions in exons 5-8 were significantly more enriched in AD patients compared to healthy control individuals (OR_MH _1.96 [95% CI 1.18-3.25]; *p_MH _= *0.009, *p*_woolf _= 0.551) (Table 4).

**Table 4:** Meta-analysis of rare *CLU *genetic variants.

	Total chromosome number		Rare genetic variants		
	AD	C	AD (%)	C (%)	OR [95% CI]
Flanders-Belgian (this study)	1698	1318	21 (1.2)	9 (0.7)	1.82 [0.8-3.39]
Lille (this study)	2610	1220	21 (0.8)	3 (0.2)	3.29 [0.98-11.05]
Toronto (this study)	612	462	6 (1.0)	2 (0.4)	2.28 [0.46-11.33]
Portugal (Guerreiro et al.) ^14^	806	470	9 (1.1)	2 (0.4)	2.64 [0.57-12.28]
UK (Guerreiro et al.) ^14^	892	1264	2 (0.2)	2 (0.2)	1.42 [0.20-10.09]
US-Caucasian (Tycko et al.) ^15^	106	86	1 (0.9)	3 (3.5)	0.26 [0.03-2.58]

**Summary**	6724	4820	60 (0.9)	21 (0.4)	1.96 [1.18-3.25]
					*p*_MH _= **0.009**
					*p*_woolf _= 0.551

Total allele counts and frequencies of rare non-synonymous substitutions affecting the *CLU *β*-*chain observed in AD patients and control individuals. *P*-values are given for fixed-effect Mantel-Haenszel test (*p*_MH_) and Woolf's test for heterogeneity (*p*_woolf_). Odds ratios are given with 95% confidence intervals; summary odds ratio is based on a fixed-effect Mantel-Haenszel meta-analysis. Significant associations are depicted in bold. Compared to our p.R338W finding, Guerreiro and colleagues^14 ^observed another non-synonymous substitution at codon 338 in 1 AD patient 5 (p.R338Q).

To assess whether the mutation frequencies of rare coding variants differed by ethnicity, we also resequenced exons 5-8 in a Caribbean Hispanic AD patient/control cohort (Table 5). This identified 2 novel benign non-synonymous substitutions affecting the CLU β-chain domain - p.E431Q and p.D331N. In addition, 3 non-synonymous substitutions found in Belgian, French and Canadian individuals were more frequently observed in Caribbean Hispanics - p.N369H, p.D380N and p.S448L (Table 5) (Figure 1).

**Table 5:** Rare non-synonymous *CLU *variants identified in Stage III Caribbean Hispanic AD cohort.

Stage III: Caribbean Hispanic AD cohort									
Gene location^a^	DNA^b^	Protein^c^	dbSNP	Total number	MAF AD	MAF C	Protein location	PolyPhen (PSIC)	SIFT
Exon 5	c.965T > C	p.P322L		2	0.0022		β-chain	possible (1.96)	tolerated (0.25)
Exon 6	c.991G > A	p.D331N		2	0.0022		β-chain	benign (0.119)	tolerated (0.28)
Exon 7	c.1291G > C	p.E431Q		1	0.0011		β-chain	benign (0.133)	tolerated (0.35)

Exon 7	c.1105A > C	p.N369H*	rs9331936	155	0.08 (84 AD)	0.07 (71 C)	β-chain	possible (1.56)	tolerated (0.00)
Exon 7	c.1138G > A	p.D380N*	rs9331938	35	0.01 (13 AD)	0.02 (22 C)	β-chain	benign(0.32)	tolerated (0.36)
Exon 8	c.1343C > T	p.S448L*	rs13494	27	0.01 (13 AD)	0.01 (14 C)	β-chain	benign(0.73)	tolerated (0.23)

Exon 6	c.1004C > T	p.T335I		1		0.0010	β-chain	benign (1.077)	tolerated (0.64)
Exon 7	c.1153G > A	p.V385I		1		0.0010	β-chain	benign (0.150)	tolerated (0.4)

^a^Gene location position according to the longest *CLU *transcript with 9 coding exons [NM_001831.2], ^b^Numbering according to CLU mRNA sequence starting at the A of the ATG translation initation codon in the reference sequence [GenBank accesion number NM_001831.2], ^c^Numbering according to CLU protein sequence [GenPept accession number NP_001822.2]; MAF = Minor Allele Frequency, calculated upon the minimum number of successful sequences. (Caribbean Hispanic cohort: 926 patient and 974 control alleles), * = 3 non-synonymous variants with intermediate frequencies (MAF = 1-5%) in patients and control individuals. Prediction of pathogenicity of missense mutations was performed using *in silico *programs PolyPhen (benign/possibly damaging/probably damaging) and SIFT (tolerated/not tolerated).

### Common *CLU *association analysis

We analyzed association in the Flanders-Belgian AD cohort with the GWA top SNP rs11136000 and 14 other SNPs in the *CLU *locus, aiming at replicating the common AD risk association with *CLU *as well as fine-mapping the common association signal. We observed significant allelic association with rs11136000 (*p *= 0.004) and 4 other SNPs (*p *< 0.05) (Table 6a). Stratification by *APOE *ε4 genotype indicated that allelic associations were only significant in the *APOE *ε4 + stratum (See table in Additional file 3). All five SNPs are positioned within the same LD block (D' between 0.12 and 1.00, r^2 ^between 0.00 and 0.96) (See figure in Additional file 4 and table in Additional file 5). Conditional logistic regression analysis showed that the 4 associated SNPs were not independent from GWA top SNP rs11136000 (data not shown).

**Table 6:** Common *CLU *associations with AD.

*(A) Allelic associations in stage I and II AD cohorts*													
		Belgian-Flanders				Lille				Toronto			
	Allele	AD (total)	C (total)	OR [95% CI]	*p*	AD (total)	C (total)	OR [95% CI]	*p*	AD (total)	C (total)	OR [95% CI]	*p*
rs867230	T	0.63 (1259)	0.59 (952)		0.074 ^(1)^	0.66 (1736)	0.62 (742)		**0.022 **^(1)^	0.60 (367)	0.63 (307)		0.288 ^(1)^
Intron 1	G	0.37 (752)	0.41 (670)	0.82 [0.70-0.96]	**0.012 **^(2)^	0.34 (904)	0.38 (456)	0.89 [0.76-1.04]	0.144 ^(2)^	0.40 (247)	0.37 (181)	1.04 [0.80-1.36]	0.771 ^(2)^

rs1532278	G	0.63 (1308)	0.61 (1006)		**< 0.01**^(1)^	0.675 (1789)	0.628 (764)		**0.004 **^(1)^	0.62 (383)	0.62 (301)		0.996 ^(1)^
Intron 3	A	0.37 (770)	0.40 (656)	0.83 [0.71-0.97]	**0.019 **^(2)^	0.325 (861)	0.372 (452)	0.83 [0.71-0.97]	**0.02 **^(2)^	0.38 (233)	0.38 (183)	1.27 [0.97-1.64]	0.078 ^(2)^

rs11136000	G	0.65 (1232)	0.61 (990)		**0.034 **^(1)^	0.66 (1707)	0.628 (764)		0.195 ^(1)^	0.61 (372)	0.63 (299)		0.645 ^(1)^
Intron 3	A	0.35 (676)	0.39 (630)	0.79 [0.68-0.93]	**0.004 **^(2)^	0.34 (875)	0.372 (452)	0.93 [0.79-1.10]	0.400 ^(2)^	0.39 (236)	0.37 (179)	1.00 [0.77-1.31]	0.986 ^(2)^

rs9331908	G	0.66 (1389)	0.69 (1161)		0.055 ^(1)^	0.62 (1712)	0.66 (841)		**0.036 **^(1)^	0.66 (416)	0.69 (338)		0.297 ^(1)^
Intron 4	A	0.34 (713)	0.31 (521)	1.18 [1.01-1.39]	**0.035 **^(2)^	0.38 (1046)	0.35 (443)	1.12 [0.96-1.30]	0.156 ^(2)^	0.34 (214)	0.31 (152)	1.20 [0.91-1.56]	0.198 ^(2)^

rs7982	C	0.63 (1228)	0.61 (1005)		0.191 ^(1)^	0.67 (1780)	0.63 (804)		**0.022 **^(1)^	0.62 (374)	0.60 (295)		0.434 ^(1)^
Exon 5	T	0.37 (734)	0.40 (657)	0.85 [0.73-0.99]	**0.037 **^(2)^	0.33 (884)	0.37 (470)	0.89 [0.76-1.03]	0.124 ^(2)^	0.38 (234)	0.36 (167)	1.16 [0.89-1.52]	0.273 ^(2)^

***(B) Meta-analysis of stage I and II AD cohorts***													
	**Allele**	**Summary OR**	***p_MH_***	***p_Bonf_***	***p_woolf'_***								

rs867230	T												
Intron 1	G	0.88 [0.79-0.97]	**0.013**	0.065	0.311								

rs1532278	G												
Intron 3	A	0.85 [0.77-0.94]	**0.001**	**0.005**	0.648								

rs11136000	G												
Intron 3	A	0.88 [0.79-0.97]	**0.013**	0.065	0.206								

rs9331908	G												
Intron 4	A	1.16 [1.04-1.28]	**0.006**	**0.030**	0.824								

rs7982	C												
Exon 5	T	0.89 [0.80-0.98]	**0.023**	0.115	0.448								

**(A) **Allele frequencies are shown with absolute numbers in brackets. ^(1) ^The first *p*-value is the 2-sided *p*-value of the unadjusted χ^2 ^statistics, ^(2) ^The second *p*-value is adjusted for age, *APOE *status and gender. Calculations of odds ratios (OR), presented with 95% confidence intervals (CI), were performed using the common allele as reference allele. **(B) **For meta-analysis, Mantel-Haenszel summary OR ratios are given with 95% CI and corresponding *p-*values (*p*_MH_) based on fixed-effects meta-analysis of age, gender and *APOE*- adjusted effect estimates of the minor allele. Nominal *p*-values (*p*_mh_, adjusted for age, gender and *APOE *effects) and Bonferroni corrected *p*-values (*p *_Bonf_) for the number of SNPs (*N *= 5) in the meta-analysis are given. *P-*values of Woolf's test for heterogeneity (*p-*_woolf_) are given.

Next, we genotyped the 5 associated SNPs in the Lille and Toronto AD cohorts and calculated allelic association with AD risk (Table 6a). Nominal significance was observed in the Lille AD cohort with 4 out of 5 SNPs and in the *APOE *ε4+ stratum of the Lille AD cohort with rs9331908 (*p *= 0.024) (See table in Additional file 6). In the Toronto cohort association was only significant in the *APOE *ε4+ stratum with rs867230 (*p *= 0.024) and rs7982 (*p *= 0.023) (See table in Additional file 6).

Meta-analysis combining data from all 3 AD cohorts, confirmed allelic association with GWA SNP rs11136000 (*p *= 0.013), but showed the strongest association after Bonferroni correction with rs1532278 (*p = *0.005) (Table 6b, figure in Additional file 7).

Next, we investigated whether the significantly increased presence of rare non-synonymous substitutions and deletion/insertions in AD patients could have driven the common association of *CLU *with AD risk. Hereto, we recalculated the allelic association in the meta-analysis after excluding the 58 carriers from the Flanders-Belgian (26), the Lille (24) and the Toronto (8) AD cohorts. Allelic associations with the 5 common SNPs remained significant (*p*-values < 0.05), indicating that the associations with the common SNPs and the rare coding variations represent two independent observations (data not shown).

## Discussion

We used an extensive resequencing approach to follow-up on the significant association of common SNPs in the *CLU *locus with increased risk for AD in 2 independent GWA studies. In the discovery stage, we used unbiased resequencing of all coding and regulatory regions of *CLU *in a Flanders-Belgian AD cohort followed by targeted sequencing of 2 independent replication cohorts of French and Canadian origin. In the Flanders-Belgian AD cohort, we obtained significant evidence for clustering of rare coding variants in exons 5 to 8, predicting 3 possible (p.T345M, p.N369H, p.T440M) and one probable (p.R338W) damaging non-synonymous substitutions and a 9-bp insertion/deletion p.T445_D447del affecting the CLU β-chain domain. Interestingly, 4 AD patients carried multiple *CLU *variations: a patient with a positive family history and onset age of 68 years harbored 2 predicted pathogenic substitutions (p.R338W, p.T345M) and three patients carried the deletion p.T445_D447del together with the benign variant p.A309T. Targeted resequencing in the Lille and Toronto AD cohorts identified predicted pathogenic substitutions (p.R338W and p.N369H) in French AD patients and the p.T445_D447del deletion in French and Canadian AD patients. Noteworthy, we observed haplotype sharing with 7 markers around the insertion/deletion site in 3 Belgian and 2 French AD patient carriers which is suggestive for a common ancestor. Partial haplotype sharing was further observed with another French and Canadian patient.

Moreover, we identified a 1-bp insertion/deletion variation p.I303NfsX13, predicting a premature stop codon after 315 amino acid residues (24 of which in β-chain), in a French patient (onset age 68 years). This frame-shift mutation would most likely produce an unstable transcript that is degraded by the nonsense mediated mRNA decay control system or an unstable C-truncated protein that is degraded within the cell, however, no patient material was available for further testing. Substitutions p.R338W and p.T345M are positioned inside the disulphide rich region which is involved in the folding of the α and β-domains into a heterodimer complex. Variant p.N369H deletes a CLU N-glycosylation signal [19], and since CLU is highly glycosylated (20-25% of its total mass comprises carbohydrates), this substitution might affect CLU processing or functioning in ligand interaction. Although absent from all Belgian and French control individuals (> 2500 control chromosomes), p.N369H was present in one Canadian control person aged 58 years, which is suggestive of an intermediate penetrance for this substitution. We also observed 3 non-synonymous substitutions with possible damaging effects (α-chain and β-chain) in both Flanders-Belgian patients and control individuals. As these variants were found in 1 to 4 control individuals only and the control group had on average a younger inclusion age than the patient group, these substitutions might represent risk factors of intermediate disease penetrance and might still contribute to AD risk. In contrast, all non-synonymous substitutions observed in control individuals were predicted to be benign and to unlikely affect CLU protein functioning, strengthening our observation that rare non-synonymous substitutions might contribute to AD risk.

Meta-analysis including all rare coding variants affecting the CLU β-chain domain observed in the discovery and replication cohorts plus those reported in 3 additional cohorts [14,15], totaling 5772 patients and controls, lent further support to an increased occurrence of rare coding variants predicted to affect the CLU β-chain in AD patients compared to healthy individuals (OR_MH _1.96 [95% CI 1.18-3.25]; *p *= 0.009). Assessing the prevalence of variations affecting the β-chain domain by targeted resequencing of *CLU *exons 5 to 8 in a Caribbean Hispanic AD cohort, detected novel benign variants while predicted pathogenic variants found in stage I and II AD cohorts were absent. Of note, predicted pathogenic variant p.N369H was more frequently observed in Hispanic patient and control individuals then in stage I and II Caucasian cohorts, suggesting this variant might be a polymorphism which is in agreement with previous reports in African-Americans [15]. Excluding p.N369H from our meta-analysis on rare variants did not alter the significance of our observation that rare coding variants affecting the CLU β-chain are more frequently observed in AD patients than control individuals (*p *= 0.001). It is possible that rare *CLU *variants contribute in a different way to disease risk in diverse populations and alternatively, that part of the genetic *CLU *variability is population-specific (e.g. Hispanic origin). Screening other and larger AD cohorts of different ethnicity and conducting functional analyses on putative pathogenic variants will help unraveling the role of these rare coding variants to AD risk. In this context it is relevant to mention that we have primarily used protein prediction algorithms that have their limitations as well. While our meta-analysis suggested that the CLU β-chain is involved in AD risk, to adequately discriminate between rare or low frequent pathogenic variants and benign polymorphisms, their pathogenicity needs to be experimentally evaluated. These experiments will have to be performed along with comparable efforts aiming at understanding the role of CLU in AD pathogenesis.

Although we identified an association of AD risk with clustering of rare coding variants affecting the CLU β chain, this association was independent from the association with rs11136000, the top GWA SNP [2,3]. Genotyping rs11136000 in the stage I and II cohorts followed by a meta- analysis showed significant association (*p *= 0.013). This association remained significant after exclusion of carriers with predicted pathogenic β-chain variants. This is in line with recent *LRRK2 *observations in which independent common risk associations were found after exclusion of carriers with known pathogenic mutations [20]. Further, high-density association fine-mapping by genotyping common SNPs throughout the *CLU *locus in the stage I cohort, identified 4 additional SNP associations, that all except one (rs9331908) were in high LD with rs11136000 (r^2 ^> 0.89) and located in the 5' *CLU *region. This association region ranges from *CLU *intron 1 to exon 5, which is only marginally overlapping with the rare variant association region in the β-chain (exon 5 to 8). Combined with the two independent association signals for common and rare *CLU *variants, this could imply two distinct mechanisms underlying AD risk.

Further genotyping of the 4 significant SNPs and combined meta-analyses on all 3 AD cohorts showed, after Bonferroni correction, the strongest evidence of association with rs1532278 (*p *= 0.005). This variant is located in an intron 3 sequence that strongly resembles a regulatory element based on sequence alignment of seven species. Also, association evidence was found for rs867230 in *CLU *intron 1, which potentially affects a MEF2 transcription factor binding site. These two novel associated common variants may represent variants of functional relevance underlying the common association signal with rs11136000 in various populations [21-23]. In agreement with other reports [14,21], we did not observe any association with common coding variants. Though we did not find association with the recently described splice site variant rs9331888 [24] in our Flanders-Belgian population (OR 1.11 [95% CI 0.94-1.31]; *p *= 0.2), our association findings do point to common variants with possible regulatory effects (rs1532278, rs867230). It is likely that a number of common and rare *CLU *variants contribute to an increased risk for AD. In addition, different mechanisms may contribute to disease: our common *CLU *associations might regulate CLU transcription while our rare *CLU *association signal results from non-synonymous variants affecting protein functioning. Whether these rare *CLU *variants represent loss or gain of function variants remains at present unclear and requires further studies.

## Conclusions

In conclusion, in-depth *CLU *resequencing showed significant clustering and association of rare coding variants with AD risk in a combined meta-analysis of 3 Caucasian AD cohorts analyzed in this study and cohorts of previous published studies. The rare coding variants are non-synonymous substitutions and insertion/deletion mutations that affect the CLU β-chain domain. While the physiological properties of the β-chain domain remain unclear, our data suggests that this protein subunit may be of interest in AD pathogenesis, and merits follow-up with detailed functional analyses. Also, in our combined AD data set, the rare coding variant association was independent of the common AD risk association signal that we fine-mapped to a more 5' region of *CLU*. The strongest association was not obtained with GWA SNP rs11136000 but with an intron 3 SNP (rs1532278) located in a sequence with high regulatory potential. Altogether, our data suggest that *CLU *may be a risk gene in which multiple rare and common variants have independent effects on AD disease susceptibility.

## Methods

### AD cohorts

#### Flanders-Belgian AD cohort

In the stage I analysis, we used as discovery sample an extended Flanders-Belgian AD cohort of 1930 subjects. A fraction of this AD cohort (*n *= 1750, 91%) contributed to the replication phase of the 2 AD GWA studies confirming AD risk association at the *CLU *locus [2,3]. Since then we extended the AD cohort dataset with newly ascertained subjects and additional genotypes. The AD patients have been ascertained at memory clinics and neurology divisions in a prospective study of neurodegenerative and vascular dementia in Flanders, the Dutch-speaking region of Belgium [25,26], and in a comparable prospective study on molecular genetics of cognitive impairment [27] using the same clinical assessments and biosampling schemes (Table 1). Each AD patient underwent a neuropsychological examination, including Mini-Mental State Examination (MMSE)[28] and structural and/or functional neuroimaging [27]. Consensus diagnosis of possible or probable AD was obtained by minimal two neurologists based on the National Institute of Neurological and Communication Disorders and Stroke-Alzheimer's Disease and Related Disorders Association (NINCDS-ADRDA) criteria [29]. Unrelated control individuals had no neurological or psychiatric antecedents or had neurological complaints or organic disease involving the central nervous system. Additional community control individuals were included after interview concerning medical and family history and a Mini Mental State Examination (MMSE) > 24 [28].

#### French and Canadian AD cohorts

In the stage II analysis, we used two independent Caucasian AD patient/control cohorts for replication of rare coding variants in *CLU *exons 5-8 by targeted resequencing. A first French AD cohort consisted of patients ascertained in the North of France (Lille AD cohort) that were diagnosed with probable AD according to DSM-III-R and NINCDS/ADRDA criteria. Healthy control individuals were from the same geographical area and were cognitively intact, had no family history of AD or DSM-III-R criteria for dementia and a MMSE ≥ 25 (Table 1) [30,31]. In a second Canadian AD cohort (Toronto AD cohort [13]), diagnoses of probable or possible AD were defined according to the NINCDS-ADRDA criteria at clinics specialized in memory disorders [29]. Subjects were classified as control individuals when they were without cognitive impairment or dementia at last visit (Table 1) [32].

#### Caribbean Hispanic AD cohort

In the stage III analysis, we initiated an assessment of the contribution of rare coding *CLU *variants in populations of different ethnicity by analyzing an AD cohort of Caribbean Hispanics ancestry for rare coding mutations by resequencing *CLU *exons 5-8 (Table 1). The diagnosis of AD was based on the NINCDS/ADRDA criteria [29,33].

### Ethical assurances

All clinical and genetic studies described in this manuscript were approved by the medical ethical committees of the respective hospital divisions and university genetic laboratories at the respective cohort sampling sites in Flanders-Belgium, France, Canada and USA. Informed consent was obtained from all participants using procedures approved by institutional review boards at each of the clinical research centers enrolling subjects.

### *CLU *resequencing

Unbiased resequencing was performed in the Flanders-Belgian AD cohort by PCR sequencing of all 9 *CLU *exons and their flanking intron-exon boundaries [NM_001831.2], the 5' and 3'UTR regions of longest transcript 1 [NM_001831.2], the 5'UTR region of transcript 2 [NM_20339.1] and regulatory elements (Table 2 and see table in Additional file 1). Primers were designed using ExonPrimer software and primer3. In stages II and III, we performed targeted resequencing of *CLU *exons 5-8 in the Lille, Toronto and Caribbean Hispanics AD cohorts (Table 3 Table 5). All sequences were analyzed by two independent researchers using Seqman, the NovoSNP software package [34] or Vector NTI software (Invitrogen).

### Statistical genetic analyses

#### Coding variant association

In stage I, an exon-by-exon approach was used to examine significance of putative clustering in AD patients of rare *CLU *non-synonymous variants with a MAF of < 0.01-0.05 (Table 4). Alleles were collapsed and overall frequency differences between AD patients and control individuals were compared using χ^2 ^statistics. Additionally, we performed a literature search identifying in PubMed all published studies that were reporting a genetic screening of *CLU *in AD patients and healthy individuals of Caucasian origin, yielding two publications describing data in three AD cohorts from UK, Portugal and US [14,15]. For the meta-analysis, we included all rare coding variants predicting non-synonymous and insertion/deletion mutations in the CLU β-chain domain. Rare variant counts in patients and control individuals were obtained for each dataset. To obtain summary ORs and 95% CI, a fixed-effect (Mantel-Haenszel) meta-analysis was performed in R using the library rmeta-version 2.16.

#### Common SNP associations

The stage I Flanders-Belgian AD cohort (*n *= 1930, Table 1) had an estimated power of 97% to detect a modest association with an odds ratio (OR) of ~1.5 and a common genetic variation with a minor allele frequency (MAF) of 0.2 [35]. Tagging SNPs were selected throughout the *CLU *locus (chr8:27.538.244 - 27.500.368; including 10 kb up- and downstream regions), using the CEPH (Centre d'Etude du Polymorphism Humain) population of HapMap (Data Rel24/phase II Nov08). SNPs with a MAF < 0.05, with a Hardy-Weinberg equilibrium (HWE) *p*-value < 0.05 or within repeat sequences (> 50%) were removed from the selection. A total of 15 SNPs including the GWA top SNP rs11136000 [2,3], were used in the stage I Flanders-Belgian AD cohort for association analysis and for fine-mapping of the association signal in the *CLU *locus (See figure in Additional file 4).

Four SNPs were genotyped by sequencing (rs7982, rs867230, rs3216167, rs11136000), the others SNPs by Sequenom MassArray^® ^assay, followed by MALDI-TOF mass spectrometry (Sequenom, Inc., Hamburg, Germany). PCR and extension primers were designed using Assay Design 3.1 Software. Genotypes were scored both automatically (MassArray Typer version 4.0) and by two researchers blind to disease status. Genotyping success rate for SNPs genotyped with Sequenom was > 95%. Inter-plate controls showed 100% concordance for all genotyped SNPs. Deviations from HWE were determined using an exact HWE test [36].

For common SNPs, differences in allele frequencies between AD patients and control individuals were tested using χ^2 ^statistics. *P*-values and odds ratios (OR) with 95% confidence intervals for the minor allele were calculated relative to the common allele and corrected for gender, onset age or age at inclusion using binary logistic regression models (Table 6a). Allelic associations were further stratified for *APOE *status i.e. presence or absence of *APOE *ε4 alleles (See table in additional file 3 and figure in Additional file 4). Statistical analyses were performed using SPSS 16.0 (SPSS, Inc., Chicago, IL). SNPs showing significant association at stage I, were genotyped in the Lille and Toronto AD cohorts and associations calculated (See table in Additional file 6). Fixed-effect (Mantel-Haenszel) meta-analysis was performed based on effect estimates of the separate cohorts adjusted for age, gender and *APOE *ε4 status in R using the library rmeta-version 2.16. Woolf's test for heterogeneity was calculated. A Bonferroni *p*-value < 0.05, corrected for the number of SNPs included in the meta-analysis (*n *= 5), was considered significant (Table 6b and see forest plots figure in Additional file 7).

## List of abbreviations

AA: Amino Acids; AD: Alzheimer's disease; AAO: age at onset; AAI: age at inclusion; CI: confidence interval; *CLU*: clusterin; GWA: genome-wide association; HWE: Hardy-Weinberg equilibrium; MAF: minor allele frequency; nt: nucleotides; OR: odds ratio; SD: standard deviation; SNP: single nucleotide polymorphism; UTR: untranslated region.

## Competing interests

The authors declare that they have no competing interests.

## Authors' contributions

KB, KS, NB and CVB were involved in the conception and design of the study, and in the analysis and interpretation of the data. SE, RV, NLB, MM, KP, PDD, J-CL, ER, RM, PSH collected the patient materials included in this study. KB, SV and JVD participated in the sequencing studies, KB performed the statistical analyses, KB and KS drafted the manuscript. J-CL, ER, RV, FP, RM, PSH, PA, SE, RV, NLB, PDD have helped to revise the manuscript for intellectual content. CVB is the principal investigator. All authors have read and approved the final manuscript.

## Supplementary Material

Additional file 1 **Synonymous *CLU *variants and *CLU *variants in 3' UTR, 5' UTR, regulatory regions, and splice sites in Flanders-Belgian AD cohort**. ^a^Gene location position according to the longest CLU transcript with 9 coding exons [NM_001831.2], ^b^Numbering according to build GRCh37/hg19 (Feb.2009), nucleotide changes given for complementary negative strand; MAF = Minor Allele Frequency, calculated upon the minimum number of successful sequences (1698 AD alleles, 1318 control alleles) **(B) **The miRanda algorithm (microRNA resource) was used to predict whether 3' UTR variants are involved in miRNA-binding [37]. TargetScan [38] and Pictar [39] were applied for prediction of miRNA binding site positions. Polymorphisms in microRNA Target Site (PolymiRTS) and Patrocles databases were used [40] to estimate miRNA binding effects of variants. **(D) **Regulatory elements (proximal promoter, OREG0018671) predicted using FirstEF and OregAnno from UCSC browser Human Mar. 2006) were sequenced, encompassing 458 nt before the translation initiation codon (transcript 1) and 457 nt downstream of coding exon 1 [NM_001831.2]. No variants were positions in conserved transcription factor binding sites (according to UCSC). **(E) **Variants near splice sites. FSPLICE, Netgene2 and SPL were used to predict possible splicing effects. Click here for file

Additional file 2 **Haplotype sharing of p.T445_D447del carriers**. Allele sharing of p.T445_D447del carriers (3 Belgian patients, 3 French and 1 Canadian AD patient) was examined by genotyping 10 short tandem repeat (STR) markers located in a 1.3 Mb region covering the *CLU *locus. ^a^Physical location of STR markers is relative to the NCBI genome build 36, DNA of two unaffected children (with inclusion age < 50 years) of Belgian Flanders-Belgian carriers allowed reconstructing haplotypes; Flanders-Belgian carriers of the insertion/deletion shared a 10-marker haplotype covering 1.3 Mb around *CLU *(shared alleles are indicated in bold), two French patient carriers shared alleles at 7 consecutive markers of this haplotype, while French patient 2 and the Canadian patient 1 only shared 3 consecutive markers with the Belgian patients. Of note, Belgian insertion/deletion carriers also carried p.A309T (Table 2), which was not detected in the French or Canadian individuals. Click here for file

Additional file 3 **Common *CLU *allelic associations in Flanders-Belgian *APOE *ε4 strata**. ^a^Gene location position according to the longest CLU transcript with 9 coding exons [NM_001831.2], ^b^Numbering according to build GRCh37/hg19 (Feb.2009), minor alleles given for complementary negative strand. Allele frequencies are shown with absolute numbers in brackets. Calculations of odds ratios, presented with 95% confidence intervals (CI), were performed using the common allele as reference allele. Nominal *p*-values were adjusted for age (onset age for patients, inclusion age for control individuals) and gender in the *APOE *subgroups. Nominally significant *p*-values are marked in bold. Click here for file

Additional file 4 **Schematic overview of *CLU *allelic associations and LD pattern in Flanders-Belgian AD cohort**. -log10 *p-*values for allelic associations of 15 common SNPs (MAF > 0.05) encompassing the *CLU *locus are given for the total cohort adjusted for age, gender and *APOE*, and for *APOE *ε4 genotype strata adjusted for age and gender (Additional file 3). Based upon 15 common SNP genotypes of stage I, the overall linkage disequilibrium (LD) plot was reconstructed with D' as LD measure drawn using the LDheatmap v0.2-8 package. The LD pattern consisted of a major LD block (12 consecutive SNPs starting from intron 3 to 3' intergenic region) and a minor LD block of 3 SNPs upstream from *CLU*. Click here for file

Additional file 5 **Linkage disequilibrium measures in stage I and II AD cohorts**. Pairwise linkage measures (D' and r^2^) are given for the associated SNPs in stage I and stage II cohorts. Click here for file

Additional file 6 **Common *CLU *allelic associations in replication AD cohorts *APOE *ε4 strata**. Allele frequencies are shown with absolute numbers in brackets, minor alleles given for complementary negative strand. Calculations of odds ratios, presented with 95% confidence intervals (CI), were performed using the common allele as reference allele. Nominal *p*-values were adjusted for age (onset age for patients, inclusion age for control individuals) and gender in the *APOE *subgroups. Nominally significant *p*-values are marked in bold. Click here for file

Additional file 7 **Forest plots of common CLU association in stage I and II AD cohorts**. Odds ratio's and 95% confidence intervals are given for stage I (Flanders-Belgian) and stage II cohorts (Lille, Toronto) separately as well as overall combining stage I and II. Click here for file
