# Supplementary material for: Both common variations and rare non-synonymous substitutions and small insertion/deletions in CLU are associated with increased Alzheimer risk
Source: Mol Neurodegener. 2012 Jan 16;7:3. doi: 10.1186/1750-1326-7-3 (PMC3296573; doi:10.1186/1750-1326-7-3)
Supplement: Additional file 1 — Synonymous CLU variants and CLU variants in 3' UTR, 5' UTR, regulatory regions, and splice sites in Flanders-Belgian AD cohort. aGene location position according to the longest CLU transcript with 9 coding exons [NM_001831.2], bNumbering according to build GRCh37/hg19 (Feb.2009), nucleotide changes given for complementary negative strand; MAF = Minor Allele Frequency, calculated upon the minimum number of successful sequences (1698 AD alleles, 1318 control alleles) (B) The miRanda algorithm (microRNA resource) was used to predict whether 3' UTR variants are involved in miRNA-binding [37]. TargetScan [38] and Pictar [39] were applied for prediction of miRNA binding site positions. Polymorphisms in microRNA Target Site (PolymiRTS) and Patrocles databases were used [40] to estimate miRNA binding effects of variants. (D) Regulatory elements (proximal promoter, OREG0018671) predicted using FirstEF and OregAnno from UCSC browser Human Mar. 2006) were sequenced, encompassing 458 nt before the translation initiation codon (transcript 1) and 457 nt downstream of coding exon 1 [NM_001831.2]. No variants were positions in conserved transcription factor binding sites (according to UCSC). (E) Variants near splice sites. FSPLICE, Netgene2 and SPL were used to predict possible splicing effects. [file 1750-1326-7-3-S1.DOC]

**Additional file 1** **Synonymous *CLU* variants and variants in 3’ UTR, 5’ UTR, regulatory regions, and splice sites in Flanders-Belgian AD cohort.**

| ***(A) Synonymous CLU variants*** | | |  |  |  |  |  |  |  |
| --- | --- | --- | --- | --- | --- | --- | --- | --- | --- |
| **Gene location^a^** | **Genomic position^b^** | **Protein** | **dbSNP** | **Total**  **number** | **MAF AD** | **MAF C** | **Protein location** |  |  |
| Exon 2 | g.27468068G>T | p.L59 | no | 2 | 0.0012 (2 AD) | - | signal peptide isoform 1 |  |  |
| Exon 2 | g.27468014G>A | p.T77 | no | 1 | 0,0006 (1 AD) | - | α-chain |  |  |
| Exon 3 | g.27466578C>T | p.Y93 | rs9331898 | 1 | 0.0006 (1 AD) | - | α-chain |  |  |
| Exon 5 | g.27462514C>T | p.H304 | no | 2 | 0.0012 (2 AD) | - | β-chain |  |  |
| Exon 8 | g.27456003G>C | p.A490 | no | 1 | 0.0006 (1 AD) | - | β-chain |  |  |
| Exon 1 | g.27472266T>C | p.C11 | no | 2 | 0.0006 (1AD) | 0.0008 (1 C) | extra AA isoform 2 |  |  |
| Exon 2 | g.27468005C>T | p.D80 | no | 3 | 0.0012 (2 AD) | 0.0008 (1 C) | α-chain |  |  |
| Exon 3 | g.27466510C>T | p.N116 | no | 5 | 0.0018 (3 AD) | 0.0015 (2 C) | α-chain |  |  |
| Exon 7 | g.27457477C>T | p.D380 | rs9331939 | 17 | 0.0047 (8 AD) | 0.0068 (9 C) | β-chain |  |  |
| ***(B) Variants in CLU 3’UTR*** | | |  |  |  |  |  |  |  |
| **Gene location^a^** | **Genomic position^b^** | **Protein** | **dbSNP** | **Total**  **number** | **MAF AD** | **MAF C** | **PolymiRTS** | **Patrocles** | **microRNA.org** |
| 3' UTR | g.27455749T>C | - | no | 1 | 0.0006 (1 AD) | - | no PolymiRTs | no motif | hsa-miR-100: no binding at variant |
| 3' UTR | g.27455514G>A | - | no | 1 | 0.0006 (1 AD) | - | no PolymiRTs | no motif |  |
| 3' UTR | g.27455399C>T | - | no | 1 | 0.0006 (1 AD) | - | no PolymiRTs | no motif |  |
| 3' UTR | g.27455351G>T | - | no | 1 | 0.0006 (1 AD) | - | no PolymiRTs | G- allele: hsa-miR-412 |  |
| 3' UTR | g.27454856C>T | - | no | 3 | 0.0018 (3 AD) | - | no PolymiRTs | G-allele: hsa-miR-885-3p |  |
| 3' UTR | g.27454855G>A | - | no | 1 | 0.0006 (1 AD) | - | no PolymiRTs | G-allele: hsa-miR-885-3p |  |
| 3' UTR | g.27454493A>G | - | no | 1 | 0.0006 (1 AD) | - | no PolymiRTs | no motif | hsa-miR-450b-5p: binding at variant |
| 3' UTR | g.27455470G>A | - | no | 4 | 0.0006 (1 AD) | 0.0023 (3 C) | no (new variant) | no motif difference | no |
| 3' UTR | g.27455412insA | - | no | 2 | 0.0006 (1 AD) | 0.0008 (1 C) | no (new variant) | no motif difference | no |
| 3' UTR | g.27455114T>C | - | rs9331942 | 80 | 0.0280 (54 AD) | 0.0210 (35 C) | no (new variant) | no motif difference | no |
| 3' UTR | g.27454957T>C | - | rs9331945 | 42 | 0.0150 (29 AD) | 0.0090 (13 C) | no (new variant) | no motif difference | no |
| 3' UTR | g.27454902G>A | - | no | 2 | 0.0006 (1 AD) | 0.0008 (1 C) | no (new variant) | no motif difference | no |
| 3' UTR | g.27454877T>C | - | rs9331947 | 154 | 0.0410 (83 AD) | 0.042 (71 C) | no (new variant) | no motif difference | hsa-miR-584: binding at variant |
| 3' UTR | g.27454788-27454791delCATT | - | rs4054912 | 2 | 0.0006 (1 AD) | 0.0008 (1 C) | no (new variant) | wt-allele: hsa-miR-569 | hsa-miR-569: binding at wt variant |
| 3' UTR | g.27454730C>T | - | no | 39 | 0.0140 (26 AD) | 0.0080 (13 C) | no (new variant) | no motif difference | no |
| 3' UTR | g.27454686A>G | - | rs9331949 | 80 | 0.0280 (52 AD) | 0.0170 (28 C) | no (new variant) | G-allele: TTTGTAGC motif | no |
| 3' UTR | g.27454575G>A | - | rs10503814 | 183 | 0.0440 (94 AD) | 0.0510 (89 C) | A-allele: miR-578, miR-526b | no motif difference | hsa-miR-876-5p: binding at variant |
| 3' UTR | g.27455748G>C | - | no | 1 | - | 0.0008 (1 C) | no (new variant) |  | hsa-miR-100: no binding at variant |
| 3' UTR | g.27455655A>C | - | no | 2 | - | 0.0015 (2 C) | no (new variant) |  | hsa-miR-1254/ hsa-miR-485-5p: binding at variant |
| 3' UTR | g.27455650C>T | - | no | 1 | - | 0.0008 (1 C) | no (new variant) |  | hsa-miR-1254/ hsa-miR-485-5p/ hsa-miR-637: binding at variant |
| 3' UTR | g.27455550G>A | - | no | 1 | - | 0.0008 (1 C) | no (new variant) |  | no |
| 3' UTR | g.27455439G>A | - | no | 2 | - | 0.0016 (2 C) | no (new variant) |  | no |
| 3' UTR | g.7455210T>C | - | no | 1 | - | 0.0008 (1 C) | no (new variant) |  | no |
| 3' UTR | g.27455194A>G | - | no | 1 | - | 0.0008 (1 C) | no (new variant) |  | no |
| 3' UTR | g.27454816T>C | - | no | 1 | - | 0.0008 (1 C) | no (new variant) |  | hsa-miR-661: no binding at variant |
| 3' UTR | g.27454668delAA | - | no | 1 | - | 0.0008 (1 C) | no (new variant) |  | hsa-miR-34a:binding at variant |
| 3' UTR | g.27454513C>T | - | no | 1 | - | 0.0008 (1 C) | no (new variant) |  | no |
| ***(C) Variants in CLU 5’UTR*** | | | |  |  |  |  |  |  |
| **Gene location^a^** | **Genomic position^b^** |  | **dbSNP** | **Total**  **number** | **MAF AD** | **MAF C** |  |  |  |
| 5' UTR transcript 2 | g.27468682C>T | - | rs885827 | 1 | 0.0006 (1 AD) | - |  |  |  |
| 5' UTR transcript 2 | g.27468866C>T | - | no | 1 | 0.0006 (1 AD) | - |  |  |  |
| 5' UTR transcript 2 | g.27472749C>T | - | no | 1 | 0.0006 (1 AD) | - |  |  |  |
| 5' UTR transcript 2 | g.27468770C>T | - | no | 2 | 0.0012 (2 AD) | - |  |  |  |
| ***(D) Variants in CLU regulatory regions*** | | | |  |  |  |  |  |  |
| **Gene location^a^** | **Genomic position^b^** |  | **dbSNP** | **Total**  **number** | **MAF AD** | **MAF C** |  |  |  |
| Regulatory region | g.27472386C>G | - | no | 2 | 0.0012 (2 AD) | - |  |  |  |
| Regulatory region | g.27472648C>T | - | no | 1 | 0.0006 (1 AD) | - |  |  |  |
| Regulatory region | g.27472749C>T | - | rs10441615 | 3 | 0.0018 (3 AD) | - |  |  |  |
| Regulatory region | g.27472047G>C | - | no | 1 | 0.0006 (1 AD) | - |  |  |  |
| Regulatory region | g.27471784C>G | - | no | 1 | 0.0006 (1 AD) | - |  |  |  |
| Regulatory region | g.27472749G>A | - | no | 2 | 0.0006 (1 AD) | 0.0008 (1 C) |  |  |  |
| Regulatory region | g.27472380-27472389dup | - | no | 8 | 0.0024 (4 AD) | 0.0030 (4 C) |  |  |  |
| Regulatory region | g.27472749G>C | - | no | 1 | - | 0.0008 (1 C) |  |  |  |
| Regulatory region | g.27472549T>G | - | no | 1 | - | 0.0008 (1 C) |  |  |  |
| Regulatory region | g.27472384C>T | - | no | 1 | - | 0.0008 (1 C) |  |  |  |
| Regulatory region | g.27471925insG | - | no | 2 | - | 0.0015 (2 C) |  |  |  |
| ***(E) Variants near splice-sites*** | | | |  |  |  |  |  |  |
| **Gene location^a^** | **Genomic position^b^** |  | **dbSNP** | **Total**  **number** | **MAF AD** | **MAF C** | **Predictions** |  |  |
| Near splice site | g.27456164-27456165delTT | - | no | 1 | 0.0006 (1 AD) | - | no splice site effects |  |  |
| Near splice site | g.27455967G>A | - | No | 1 | 0.0006 (1 AD) | - | no splice site effects |  |  |
| Near splice site | g.27456173C>T | - | No | 1 | 0.0006 (1 AD) | - | no splice site effects |  |  |
| Near splice site | g.27467985G>A | - | rs9331893 | 3 | 0.0012 (2 AD) | 0.0008 (1 C) | no splice site effects |  |  |
