# Supplementary material for: Both common variations and rare non-synonymous substitutions and small insertion/deletions in CLU are associated with increased Alzheimer risk
Source: Mol Neurodegener. 2012 Jan 16;7:3. doi: 10.1186/1750-1326-7-3 (PMC3296573; doi:10.1186/1750-1326-7-3)
Supplement: Additional file 2 — Haplotype sharing of p.T445_D447del carriers. Allele sharing of p.T445_D447del carriers (3 Belgian patients, 3 French and 1 Canadian AD patient) was examined by genotyping 10 short tandem repeat (STR) markers located in a 1.3 Mb region covering the CLU locus. aPhysical location of STR markers is relative to the NCBI genome build 36, DNA of two unaffected children (with inclusion age < 50 years) of Belgian Flanders-Belgian carriers allowed reconstructing haplotypes; Flanders-Belgian carriers of the insertion/deletion shared a 10-marker haplotype covering 1.3 Mb around CLU (shared alleles are indicated in bold), two French patient carriers shared alleles at 7 consecutive markers of this haplotype, while French patient 2 and the Canadian patient 1 only shared 3 consecutive markers with the Belgian patients. Of note, Belgian insertion/deletion carriers also carried p.A309T (Table 2), which was not detected in the French or Canadian individuals. [file 1750-1326-7-3-S2.DOC]

**Additional file 2 Haplotype sharing of p.T445_D447del carriers.**

| **p.T445_D447del carriers** | | **Flanders-Belgium** | | | | | | | | | | **Lille** | | | | | | **Toronto** | |
| --- | --- | --- | --- | --- | --- | --- | --- | --- | --- | --- | --- | --- | --- | --- | --- | --- | --- | --- | --- |
| **Marker** | **Physical location (bp)^a^** | **Belgian patient 1** |  | **Unaffected child of Belgian patient 1** |  | **Belgian patient 2** |  | **Belgian patient 3** |  | **Unaffected child of Belgian patient 3** |  | **French patient 1** |  | **French patient 2** |  | **French patient 3** |  | **Canadian patient 1** |  |
| D8S1809 | 28247294 | **206** | 218 | **206** | 214 | **206** | **206** | **206** | **206** | **206** | 218 | **206** | 218 | 206 | 206 | **206** | 218 | 200 | 218 |
| D8S1820 | [28053551](http://genome.ucsc.edu/cgi-bin/hgTracks?hgsid=172091361&db=hg18&position=chr8%3A28053551-28053591) | **108** | 102 | **108** | 108 | **108** | **108** | **108** | 100 | **108** | 100 | **108** | **108** | 100 | 100 | **108** | **108** | 172 | 188 |
| 11_17GT | [28003919](http://genome.ucsc.edu/cgi-bin/hgTracks?hgsid=172091361&db=hg18&position=chr8%3A28003919-28003952) | **411** | 421 | **411** | 421 | **411** | 421 | **411** | 425 | **411** | 431 | **411** | 419 | 425 | 425 | **411** | 421 | 419 | 419 |
| 10_31TA | [27779014](http://genome.ucsc.edu/cgi-bin/hgTracks?hgsid=172091361&db=hg18&position=chr8%3A27779014-27779076) | **383** | 395 | **383** | 373 | **383** | 387 | **383** | **383** | **383** | 391 | **383** | 387 | 387 | 389 | **383** | 387 | 385 | 387 |
| D8S500 | 27574174 | **172** | 176 | **172** | 180 | **172** | 180 | **172** | **172** | **172** | 172 | **172** | 184 | **172** | **172** | **172** | 184 | 172 | 188 |
| 9_16TG | [27551751](http://genome.ucsc.edu/cgi-bin/hgTracks?hgsid=172091361&db=hg18&position=chr8%3A27551751-27551783) | **347** | 363 | **347** | 363 | **347** | 363 | **347** | **347** | **347** | 347 | **347** | **347** | **347** | 363 | **347** | **347** | **347** | 363 |
|  |  |  |  |  |  |  |  |  |  |  |  |  |  |  |  |  |  |  |  |
| GDB211528 | [27404134](http://genome.ucsc.edu/cgi-bin/hgTracks?hgsid=172091361&db=hg18&position=chr8%3A27404134-27404188) | **119** | **119** | **119** | 121 | **119** | 101 | **119** | 115 | **119** | 119 | **119** | 121 | **119** | **119** | **119** | 121 | 110 | **119** |
| 6_24TG | [27292036](http://genome.ucsc.edu/cgi-bin/hgTracks?hgsid=172091361&db=hg18&position=chr8%3A27292036-27292084) | **316** | 302 | **316** | 306 | **316** | 304 | **316** | 306 | **316** | 306 | 304 | 308 | 308 | 312 | 304 | 308 | 314 | **316** |
| 3_21AC | [27170894](http://genome.ucsc.edu/cgi-bin/hgTracks?hgsid=172091361&db=hg18&position=chr8%3A27170894-27170936) | **136** | 148 | **136** | 148 | **136** | 130 | **136** | 142 | **136** | 144 | 136 | 152 | 144 | 152 | 136 | 152 | 146 | 148 |
| 1_21AC | [26982184](http://genome.ucsc.edu/cgi-bin/hgTracks?hgsid=172091361&db=hg18&position=chr8%3A26982184-26982225) | **203** | 201 | **203** | 201 | **203** | 207 | **203** | 201 | **203** | 201 | 209 | 213 | 205 | 207 | 209 | 213 | 201 | 205 |
