# Supplementary material for: Both common variations and rare non-synonymous substitutions and small insertion/deletions in CLU are associated with increased Alzheimer risk
Source: Mol Neurodegener. 2012 Jan 16;7:3. doi: 10.1186/1750-1326-7-3 (PMC3296573; doi:10.1186/1750-1326-7-3)
Supplement: Additional file 3 — Common CLU allelic associations in Flanders-Belgian APOE ε4 strata. aGene location position according to the longest CLU transcript with 9 coding exons [NM_001831.2], bNumbering according to build GRCh37/hg19 (Feb.2009), minor alleles given for complementary negative strand. Allele frequencies are shown with absolute numbers in brackets. Calculations of odds ratios, presented with 95% confidence intervals (CI), were performed using the common allele as reference allele. Nominal p-values were adjusted for age (onset age for patients, inclusion age for control individuals) and gender in the APOE subgroups. Nominally significant p-values are marked in bold. [file 1750-1326-7-3-S3.DOC]

**Additional file 3 Common *CLU* allelic associations in Flanders-Belgian *APOE* ε4 strata.**

| **Flanders-Belgian AD cohort** | | | | ***APOE* ε4 negative subgroup** | | | | ***APOE* ε4 positive subgroup** | | | |
| --- | --- | --- | --- | --- | --- | --- | --- | --- | --- | --- | --- |
| **db SNP** | **Gene location^a^** | **Genomic**  **position^b^** | **Minor Allele** | **MAF AD (total)** | **MAF C (total)** | ***p*-value** | **OR**  **[95% CI]** | **MAF AD (total)** | **MAF C (total)** | ***p-* value** | **OR**  **[95% CI]** |
| rs507341 | Upstream | g.27481734 | G | 0.28 (275) | 0.29 (347) | 0.59 | 0.95  [0.77-1.16] | 0.29 (325) | 0.29 (131) | 0.67 | 0.94  [0.72-1.24 |
| rs569205 | Upstream | g.27477269 | A | 0.38 (367) | 0.38 (456) | 0.97 | 1.00  [0.82-1.21] | 0.38 (423) | 0.37 (169) | 0.82 | 0.97  [0.75-1.25] |
| rs538181 | Upstream | g.[27476815](http://genome.ucsc.edu/cgi-bin/hgTracks?hgsid=223626119&db=hg19&position=chr8%3A27476815-27476815) | C | 0.44 (430) | 0.43 (520) | 0.71 | 1.04  [0.86-1.25] | 0.41 (463) | 0.41 (186) | 0.50 | 0.92  [0.72-1.18] |
| **rs867230** | Intron 1 | g.[27468503](http://genome.ucsc.edu/cgi-bin/hgTracks?hgsid=223626119&db=hg19&position=chr8%3A27468503-27468503) | G | 0.39 (367) | 0.41 (477) | 0.21 | 0.88  [0.73-1.07] | 0.38 (416) | 0.42 (184) | **0.01** | 0.72  [0.56-0.93] |
| **rs1532278** | Intron 3 | g.[27466315](http://genome.ucsc.edu/cgi-bin/hgTracks?hgsid=223626119&db=hg19&position=chr8%3A27466315-27466315) | A | 0.38 (365) | 0.40 (467) | 0.35 | 0.91  [0.75-1.11] | 0.36 (403) | 0.41 (181) | **0.007** | 0.71  [0.54-0.91] |
| **rs11136000** | Intron 3 | g.27464519 | A | 0.36 (319) | 0.39 (442) | 0.20 | 0.88  [0.72-1.07] | 0.35 (356) | 0.40 (179) | **0.002** | 0.67  [0.52-0.83] |
| **rs9331908** | Intron 4 | g.27463618 | A | 0.33 (320) | 0.31 (369) | 0.27 | 1.12  [0.92-1.37] | 0.35 (392) | 0.32 (141) | **0.04** | 1.32  [1.01-1.71] |
| **rs7982** | Exon 5 | g.27462731 | T | 0.39 (358) | 0.39 (466) | 0.69 | 0.96  [0.79-1.17] | 0.36 (374) | 0.41 (182) | **0.004** | 0.68  [0.53-0.88] |
| rs3216167 | Intron 6 | g.[27461773](http://genome.ucsc.edu/cgi-bin/hgTracks?hgsid=223626119&db=hg19&position=chr8%3A27461773-27461773) | delT | 0.28 (253) | 0.28 (330) | 0.82 | 1.03  [0.83-1.27] | 0.29 (313) | 0.30 (131) | 0.29 | 1.16  [0.88-1.53] |
| rs9331930 | Intron 6 | g.[27458294](http://genome.ucsc.edu/cgi-bin/hgTracks?hgsid=223626119&db=hg19&position=chr8%3A27458294-27458294) | C | 0.29 (282) | 0.29 (348) | 0.79 | 1.03  [0.84-1.27] | 0.28 (317) | 0.25 (114) | 0.21 | 1.19  [0.90-1.58] |
| rs3087554 | 3' UTR | g.[27455442](http://genome.ucsc.edu/cgi-bin/hgTracks?hgsid=223626119&db=hg19&position=chr8%3A27455442-27455442) | G | 0.18 (172) | 0.17 (207) | 0.93 | 1.01  [0.79-1.30] | 0.18 (199) | 0.19  (87) | 0.57 | 1.10  [0.9-1.50] |
| rs17057444 | Downstream | g.[27453793](http://genome.ucsc.edu/cgi-bin/hgTracks?hgsid=223626119&db=hg19&position=chr8%3A27453793-27453793) | C | 0.05 (46) | 0.05 (55) | 0.28 | 1.29  [0.82-2.02] | 0.04 (47) | 0.06  (29) | 0.18 | 0.69  [0.40-1.19] |
| rs2279591 | Downstream | g.[27453763](http://genome.ucsc.edu/cgi-bin/hgTracks?hgsid=223626119&db=hg19&position=chr8%3A27453763-27453763) | A | 0.26 (251) | 0.26 (310) | 0.80 | 0.97  [0.78-1.21] | 0.26 (293) | 0.27 (122) | 0.95 | 1.01  [0.77-1.33] |
| rs17057438 | Downstream | g.[27450073](http://genome.ucsc.edu/cgi-bin/hgTracks?hgsid=223626119&db=hg19&position=chr8%3A27450073-27450073) | T | 0.33 (322) | 0.33 (392) | 0.26 | 1.06  [0.87-1.30] | 0.34 (375) | 0.31 (141) | 0.07 | 1.27  [0.98-1.66] |
| rs10503813 | Downstream | g.[27446900](http://genome.ucsc.edu/cgi-bin/hgTracks?hgsid=223626119&db=hg19&position=chr8%3A27446900-27446900) | C | 0.28 (274) | 0.28 (340) | 0.76 | 1.03  [0.84-1.28] | 0.28 (316) | 0.25 (116) | 0.22 | 1.19  [0.90-1.57] |
