# Supplementary figures and images for: Both common variations and rare non-synonymous substitutions and small insertion/deletions in CLU are associated with increased Alzheimer risk
Source: Mol Neurodegener. 2012 Jan 16;7:3. doi: 10.1186/1750-1326-7-3 (PMC3296573; doi:10.1186/1750-1326-7-3)

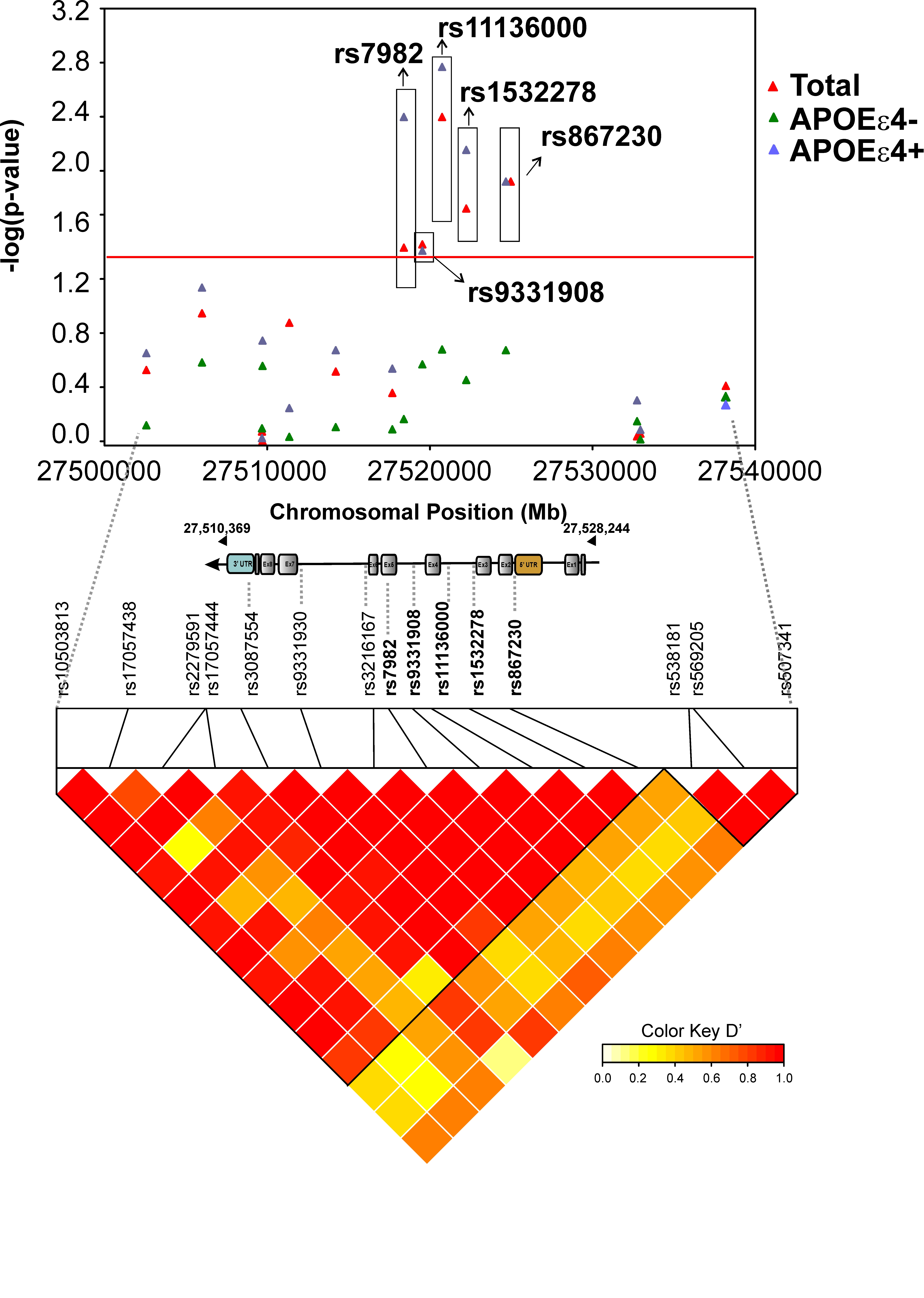

Supplement: Additional file 4 — Schematic overview of CLU allelic associations and LD pattern in Flanders-Belgian AD cohort. -log10 p-values for allelic associations of 15 common SNPs (MAF > 0.05) encompassing the CLU locus are given for the total cohort adjusted for age, gender and APOE, and for APOE ε4 genotype strata adjusted for age and gender (Additional file 3). Based upon 15 common SNP genotypes of stage I, the overall linkage disequilibrium (LD) plot was reconstructed with D' as LD measure drawn using the LDheatmap v0.2-8 package. The LD pattern consisted of a major LD block (12 consecutive SNPs starting from intron 3 to 3' intergenic region) and a minor LD block of 3 SNPs upstream from CLU. [file 1750-1326-7-3-S4.JPEG]

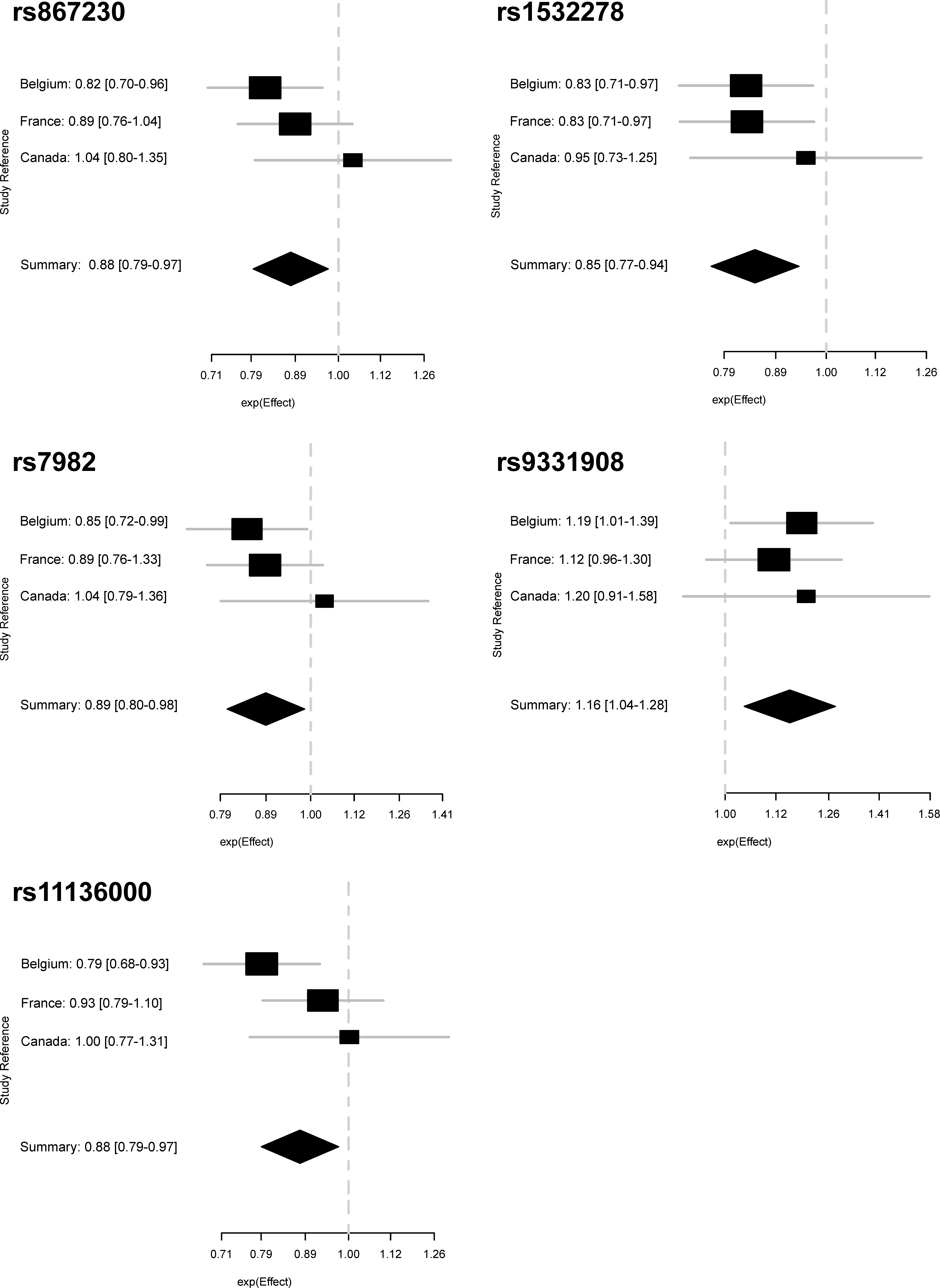

Supplement: Additional file 7 — Forest plots of common CLU association in stage I and II AD cohorts. Odds ratio's and 95% confidence intervals are given for stage I (Flanders-Belgian) and stage II cohorts (Lille, Toronto) separately as well as overall combining stage I and II. [file 1750-1326-7-3-S7.JPEG]
