# Supplementary material for: Both common variations and rare non-synonymous substitutions and small insertion/deletions in CLU are associated with increased Alzheimer risk
Source: Mol Neurodegener. 2012 Jan 16;7:3. doi: 10.1186/1750-1326-7-3 (PMC3296573; doi:10.1186/1750-1326-7-3)
Supplement: Additional file 5 — Linkage disequilibrium measures in stage I and II AD cohorts. Pairwise linkage measures (D' and r2) are given for the associated SNPs in stage I and stage II cohorts. [file 1750-1326-7-3-S5.DOC]

**Additional file 5 Linkage disequilibrium measures in stage I and II AD cohorts.**

| **Stage I: Flanders-Belgian AD cohort**  D’ measures | | | | | | r^2^ measures |  |  |  |  |  |
| --- | --- | --- | --- | --- | --- | --- | --- | --- | --- | --- | --- |
|  | rs7982 | rs9331908 | rs11136000 | rs1532278 | rs867230 |  | rs7982 | rs9331908 | rs11136000 | rs1532278 | rs867230 |
| rs7982 | 1 | 0.984 | 0.983 | 0.975 | 0.967 | rs7982 | 1 | 0.289 | 0.960 | 0.948 | 0.886 |
| rs9331908 | - | 1 | 0.993 | 0.994 | 0.991 | rs9331908 | - | 1 | 0.292 | 0.292 | 0.313 |
| rs11136000 | - | - | 1 | 0.971 | 0.966 | rs11136000 | - | - | 1 | 0.933 | 0.887 |
| rs1532278 | - | - | - | 1 | 0.959 | rs1532278 | - | - | - | 1 | 0.874 |
| rs867230 | - | - | - | - | 1 | rs867230 | - | - | - | - | 1 |
| **Stage II: Lille AD cohort**  D’ measures | | | |  |  | r^2^ measures |  |  |  |  |  |
|  | rs7982 | rs9331908 | rs11136000 | rs1532278 | rs867230 |  | rs7982 | rs9331908 | rs11136000 | rs1532278 | rs867230 |
| rs7982 | 1 | 0.994 | 0.927 | 0.977 | 0.949 | rs7982 | 1 | 0.300 | 0.850 | 0.944 | 0.844 |
| rs9331908 | - | 1 | 0.918 | 0.991 | 0.975 | rs9331908 |  | 1 | 0.256 | 0.297 | 0.308 |
| rs11136000 | - | - | 1 | 0.908 | 0.960 | rs11136000 | - | - | 1 | 0.810 | 0.756 |
| rs1532278 | - | - | - | 1 | 0.960 | rs1532278 | - | - | - | 1 | 0.854 |
| rs867230 | - | - | - | - | 1 | rs867230 | - | - | - | - | 1 |
| **Stage II: Toronto AD cohort**  D’ measures | | | |  |  | r^2^ measures |  |  |  |  |  |
|  | rs7982 | rs9331908 | rs11136000 | rs1532278 | rs867230 |  | rs7982 | rs9331908 | rs11136000 | rs1532278 | rs867230 |
| rs7982 | 1 | 1 | 1 | 0.996 | 0.966 | rs7982 | 1 | 0.296 | 0.983 | 0.979 | 0.894 |
| rs9331908 | - | 1 | 1 | 1 | 0.989 | rs9331908 | - | 1 | 0.298 | 0.293 | 0.297 |
| rs11136000 | - | - | 1 | 0.996 | 0.959 | rs11136000 | - | - | 1 | 0.964 | 0.891 |
| rs1532278 | - | - | - | 1 | 0.971 | rs1532278 | - | - | - | 1 | 0.896 |
| rs867230 | - | - | - | - | 1 | rs867230 | - | - | - | - | 1 |
