# Supplementary material for: Both common variations and rare non-synonymous substitutions and small insertion/deletions in CLU are associated with increased Alzheimer risk
Source: Mol Neurodegener. 2012 Jan 16;7:3. doi: 10.1186/1750-1326-7-3 (PMC3296573; doi:10.1186/1750-1326-7-3)
Supplement: Additional file 6 — Common CLU allelic associations in replication AD cohorts APOE ε4 strata. Allele frequencies are shown with absolute numbers in brackets, minor alleles given for complementary negative strand. Calculations of odds ratios, presented with 95% confidence intervals (CI), were performed using the common allele as reference allele. Nominal p-values were adjusted for age (onset age for patients, inclusion age for control individuals) and gender in the APOE subgroups. Nominally significant p-values are marked in bold. [file 1750-1326-7-3-S6.DOC]

**Additional file 6 Common *CLU* allelic associations in replication AD cohorts *APOE* ε4 strata.**

| **Lille AD cohort** | |  | ***APOE* ε4 negative subgroup** | | | | ***APOE* ε4 positive subgroup** | | | |
| --- | --- | --- | --- | --- | --- | --- | --- | --- | --- | --- |
| **dbSNP** | **Location** | **Minor Allele** | **MAF AD (total)** | **MAF C (total)** | ***p*-value** | **OR**  **[95% CI]** | **MAF AD (total)** | **MAF C (total)** | ***p*-value** | **OR**  **[95% CI]** |
| rs867230 | Intron 1 | G | 0.37 (401) | 0.38 (366) | 0.50 | 0.94  [0.78-1.13] | 0.30 (439) | 0.38 (83) | 0.07 | 0.77  [0.58-1.03] |
| rs1532278 | Intron 3 | A | 0.34 (375) | 0.37 (359) | 0.10 | 0.86  [0.72-1.03] | 0.31 (477) | 0.37 (87) | 0.08 | 1.18  [0.88-1.60] |
| rs11136000 | Intron 3 | A | 0.36 (380) | 0.36 (307) | 0.805 | 0.98  [0.81-1.18] | 0.32 (491) | 0.36 (76) | 0.50 | 1.02  [1.0-1.04] |
| rs9331908 | Intron 4 | A | 0.35 (392) | 0.35 (354) | 0.861 | 1.02  [0.85-1.22] | 0.40 (644) | 0.34 (86) | **0.024** | 1.38  [1.04-1.83] |
| rs7982 | Exon 5 | T | 0.36 (386) | 0.37 (375) | 0.365 | 0.92  [0.77-1.10] | 0.31 (488) | 0.36 (89) | 0.141 | 0.81  [0.61-1.07] |
| **Toronto AD cohort** | |  | ***APOE* ε4 negative subgroup** | | | | ***APOE* ε4 positive subgroup** | | | |
| **dbSNP** | **Location** | **Minor Allele** | **MAF AD (total)** | **MAF C (total)** | ***p*-value** | **OR**  **[95% CI]** | **MAF AD (total)** | **MAF C (total)** | ***p*-value** | **OR**  **[95% CI]** |
| rs867230 | Intron 1 | G | 0.34  (91) | 0.38 (137) | 0.169 | 0.78  [0.56-1.11] | 0.45 (153) | 0.33 (39) | **0.024** | 1.67  [1.07-2.59] |
| rs1532278 | Intron 3 | A | 0.33 (91) | 0.40 (141) | 0.089 | 0.75  [0.53-1.05] | 0.41 (139) | 0.32 (37) | 0.094 | 1.47  [0.94-2.32] |
| rs11136000 | Intron 3 | A | 0.34 (90) | 0.39 (139) | 0.145 | 0.78  [0.55-1.09] | 0.42 (143) | 0.31 (35) | 0.051 | 1.58  [1.00-2.51] |
| rs9331908 | Intron 4 | A | 0.36 (98) | 0.31 (113) | 0.185 | 1.26  [0.90-1.78] | 0.32 (112) | 0.30 (36) | 0.645 | 1.11  [0.70-1.77] |
| rs7982 | Exon 5 | T | 0.34 (87) | 0.39 (134) | 0.184 | 0.79  [0.56-1.12] | 0.42 (143) | 0.29 (32) | **0.023** | 1.73  [1.08-2.77] |
